# Gasocrine hypothesis – a potential supplement to cell theory

**DOI:** 10.3389/abp.2025.15465

**Published:** 2025-12-10

**Authors:** Savani Anbalagan

**Affiliations:** Institute of Molecular Biology and Biotechnology, Faculty of Biology, Adam Mickiewicz University, Poznań, Poland

**Keywords:** gasocrine signaling, gasoreceptor, gaseous signaling molecule, gas sensing, cell theory, gasocrinology, riboceptor, quorum sensing

## Abstract

Biology textbooks lack precise terms to describe oxygen-based inter-organismal signaling between oxygen-producing and aerobic organisms. To address this gap, I recently proposed the concept of gasocrine signaling, which encompasses all signaling mediated by gaseous molecules and gasoreceptors within and between cells, organisms, and even abiotic factors. Given the fundamental importance of gaseous molecules for life, I propose the gasocrine hypothesis: all cells require gasocrine signaling. This hypothesis can be divided into three sub-hypotheses: First, all living organisms composed of one or more cells require gasocrine signaling to sense, communicate, grow, and propagate. Second, gasocrine signaling mediated via gasoreceptor proteins (or yet to be identified gas-sensing riboceptors) is the most essential cellular and inter-organismal signaling. Third, acellular entities arising from or replicating in pre-existing cells require gasocrine signaling. I propose several potential experiments to falsify these hypotheses. If they withstand falsification, the gasocrine hypothesis would supplement cell theory. It would also provide a novel framework for understanding fundamental biological principles, cellular and organismal communication, and the role of abiotic factors. Furthermore, it establishes the foundation for the emerging field of gasocrinology, which is critical in the context of a changing environment.

## If there is a term “cytokine signaling” for cytokines, what is the term for gases?

A fundamental question in biology is how organelles, cells, and organisms sense their environment and communicate. Communication is typically initiated by ligand-receptor interactions, which trigger various signaling pathways. Protein-based receptors for light, temperature, amino acids, nutritional factors, and environmental chemicals have been experimentally identified in numerous model organisms (Roberts and Kruchten, 2016). Several hundred protein-based ligands and receptors have been reported in humans and zebrafish (Ramilowski et al., 2015; Chodkowski et al., 2023). However, gases are a relatively overlooked ligand class.

Gases are synthesized not only by organisms, but also by abiotic elements, man-made objects, and machines (West, 2022). *In vivo* gas sensing has been largely ascribed to: 1) “gas sensor” proteins that use gas as a substrate for its enzymatic activity; 2) proteins that undergo gas-dependent post-translational modifications; 3) metalloproteins that exhibit structural and activity changes due to interaction with the gases’ reactive species; and 4) tissues or specialized cellular structures that sense the gas and act as “chemoreceptor organs” (Aono, 2017; Bishop and Ratcliffe, 2020; Hammarlund et al., 2020; Wareham et al., 2018). However, unlike the well-accepted terminology of “receptor” for essential amino acids or factors such as temperature, the term “receptor” for gases is restricted to nitric oxide (NO), ethylene, and some volatile chemical compounds (Arnold et al., 1977; Horst et al., 2019; Binder, 2020).

Ethylene and NO receptors are well known in ripening processes in plants and vasodilation in mammals, respectively (Bakshi et al., 2015; Ignarro and Freeman, 2017). Such receptors bind the gaseous molecule in one domain via metal cofactors, such as heme or a copper ion, and signal through an additional domain (Binder, 2020; Delgado-Nixon et al., 2000; Gray et al., 2004). Similarly, some proteins can also interact with freely diffusing gases without the need for metal cofactors. For example, hemoglobin binds to carbon dioxide (CO_2_) through carbamate formation at specific amino groups (Faggiano et al., 2022). Recently, I proposed the unifying term “gasoreceptor” to refer to all such gas-binding proteins, regardless of their signaling domain or function. If the “binding” of gaseous molecules (or lack thereof) can trigger a cellular signal or response, then the protein is considered as a gasoreceptor (Anbalagan, 2025a; 2024a).

A gasoreceptor is a protein that directly detects and responds to gaseous molecules or gasotransmitters, triggering cellular signals or responses. The term gasoreceptor can unite diverse researchers who work on diverse classes of gas-activated or inhibited proteins. This raises the question of how to describe gas-gasoreceptor-based signaling? Should it be simply referred to as “gas signaling”? If so, would it also encompass all exogenous and endogenous gaseous molecules in a cell?

The term “cytokine” was first proposed in 1974 when research on lymphokines produced by immune cells revealed a general biological phenomenon (Cohen et al., 1974). Today, the term “cytokine” refers to numerous proteins produced by immune cells and various other cell types. Signaling by cytokines via cytokine receptors is simply referred to as “cytokine signaling.” However, unlike cytokine signaling, NO signaling in animals and ethylene signaling in plants are referred as gasotransmitter signaling and phytohormone signaling, respectively (Binder, 2020; Wang, 2014). This suggests a barrier in terminology even among researchers of gas signaling. Furthermore, NO and ethylene are not the only major gaseous signaling molecules.

Oxygen (O_2_) is another major gaseous molecule essential for cellular metabolism and acts as a substrate for numerous O_2_-dependent enzymes (Cogliati et al., 2021; Li et al., 2023). Despite significant research on hypoxia and O_2_ sensing mechanisms, O_2_ is not yet widely accepted as a direct gaseous signaling molecule in animal and plant gas signaling studies (Hammarlund et al., 2020; Ratcliffe and Keeley, 2025; Sies et al., 2022; Weits et al., 2021). In prokaryotic and eukaryotic model organisms, O_2_ is sensed by two types of sensors: O_2_ sensors, which require O_2_ as a substrate, and O_2_-binding proteins, which act as gasoreceptors without metabolizing it (Hammarlund et al., 2020; Bishop and Ratcliffe, 2020; Anbalagan, 2024b). Examples for the latter include the *Escherichia coli* Direct Oxygen Sensor (DosP)-phosphodiesterase, the *Leishmania major* soluble adenylate cyclase (HemAc-Lm), and the *Caenorhabditis elegans* soluble guanylate cyclase (GCY-35) (Delgado-Nixon et al., 2000; Gray et al., 2004; Sen Santara et al., 2013). These proteins can bind and sense O_2_ and trigger cellular signaling events and changes in behavior.

Proteins such as DosP or GCY-35 are often referred to as O_2_ sensors rather than receptors. This terminology can lead to confusion between O_2_ sensors that use O_2_ as a substrate and those that function as genuine O_2_ gasoreceptors by detecting O_2_ without metabolizing it. Recently, I also proposed that proteins traditionally known for O_2_ transport, such as hemoglobin and orthologs must be also considered as microenvironment-dependent O_2_ gasoreceptors (Anbalagan, 2025a). Collectively, these findings and proposal suggest that gas-gasoreceptor-based signaling occurs between O_2_-producing organisms and diverse aerobic organisms. O_2_ is not classified as a gasotransmitter in microbes or mammals, nor is it recognized as a phytohormone in plants; therefore, O_2_ signaling is not referred to as gasotransmitter or phytohormone signaling (Anbalagan, 2024b; Salvi et al., 2021; Wang, 2014). However, this raises the question of how to define such gas-mediated inter-organismal signaling–especially when teaching environmentally conscious students (Hammarlund et al., 2020; Bishop and Ratcliffe, 2020; Weits et al., 2021).

In light of the diverse roles of gaseous molecules in biological signaling, a key question remains: should we adopt a broad term such as “endogenous or exogenous/environmental gaseous signaling of biotic origin”? Or should the definition of “pheromones” be expanded to include O_2_ as a pheromone for aerobic organisms? (Agosta, 1992) Conversely, should we restrict the term “gas-mediated signaling” to gases such as NO or ethylene? In a changing environment, there is an urgent need for the scientific community to develop or embrace a unified terminology and conceptual framework. Such a framework should encompass the diverse gaseous signaling molecules and pathways across different cells, organisms and contexts.

## Gasocrine signaling–a comprehensive term and framework for gas signaling

A new term “gasocrine,” was recently proposed to encompass diverse biological and non-biological processes that produce and release gases (Anbalagan, 2024a). Gasocrine signaling refers specifically to cellular signaling mediated by gaseous molecules binding to gasoreceptors, which trigger cellular signals or responses. These gasoreceptors detect gaseous molecules in their molecular form, distinguishing gasocrine signaling from effects caused by reactive species (Anbalagan, 2024a; 2024c).

**FIGURE 1:**
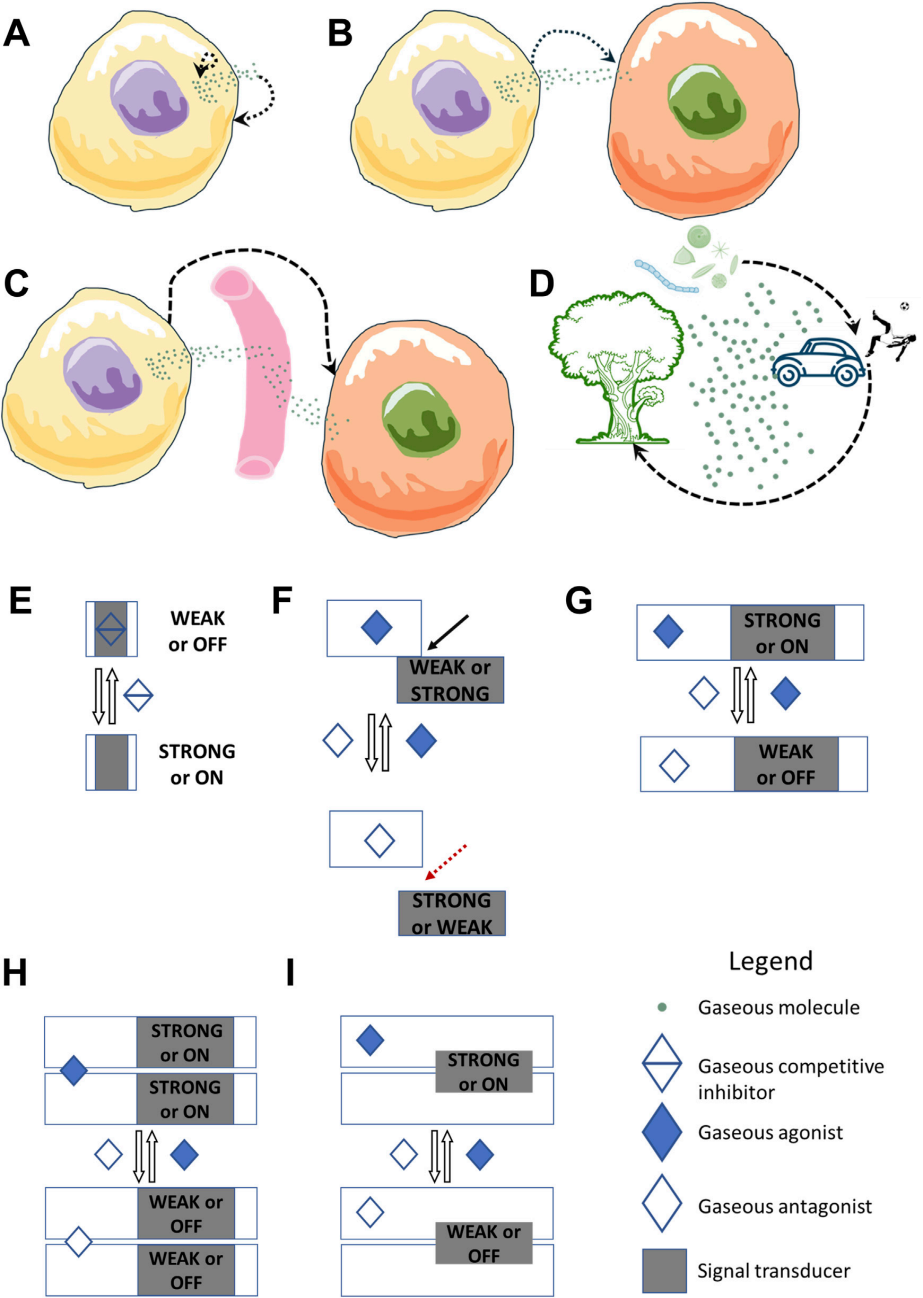
Gasocrine signaling–a comprehensive framework for gas signaling. Gasocrine signaling encompasses both endogenous gases produced by cells and exogenous gases released from environmental or abiotic sources. Gasocrine signaling unifies all signaling events mediated by gaseous molecules and their gasoreceptors, encompassing various modes: **(A)** autocrine, **(B)** paracrine, **(C)** endocrine, and **(D)** inter-organismal signaling across ecosystems and man-made machines, and potentially even at a planetary level. Gasoreceptors can be proteins with various activities, such as enzymes, transcription factors, ion channels, anion exchangers, or small or truncated gas-binding proteins without additional allosteric sites. Gasoreceptors can bind gases reversibly and function in different signal transduction systems (STS), some of which are shown here. **(E)** A gasoreceptor in proto-component STS has mutually exclusive input and output domains for gas binding and signaling, respectively. It lacks additional allosteric sites. Binding of gases as competitive inhibitors in proto-gasoreceptors will result in an absence of a cellular signaling response. **(F)** A gasoreceptor in split-component STS consists of at least two proteins. One protein has an input domain that binds and detects gases, and the other protein has an output domain that triggers the signaling response. Gas binding leads to changes in the interaction and activity of the signal transducer. **(G)** A gasoreceptor in one-component STS is a protein that contains both a gas-binding input domain and an output domain. Gas binding at the input domain can promote or inhibit output domain activity. **(H,I)** In co-component STS, a gasoreceptor can be a dimeric (or oligomeric) protein, where either the input domain or the output domains are shared between monomeric subunits. Examples include plant ethylene gasoreceptor kinases and mammalian nitric oxide gasoreceptor sGC. Gas binding will lead to changes in the activity of the output domain. The black dashed line with arrows indicates potential interactions or feedback loops. The black line with an arrow and the red dashed line with an arrow indicate the formation and separation of the complex.

Gasocrine signaling covers gases produced endogenously by cells as well as those released from environmental or abiotic sources. It unifies all signaling events mediated by gaseous signaling molecules and their gasoreceptors, encompassing various modes: autocrine, paracrine, endocrine, and inter-organismal signaling across ecosystems, potentially even at a planetary level (Figures 1A–D).

Well-established examples of gasocrine signaling include NO as a gasotransmitter in mammals and ethylene as a phytohormone in plants. Beyond these, many gases - such as O_2_, CO_2_, hydrogen sulfide (H_2_S), hydrogen cyanide (HCN), methane (CH_4_), nitrogen (N_2_), ammonia (NH_3_) and xenon (Xe) are also potential gasocrine signaling molecules, though their gasoreceptors remain less characterized.

Due to limited knowledge of gasoreceptors, systemic identification and characterization of their molecular identities, spatio-temporal localization, and activation patterns are urgently needed. The potentially broad diversity of gasoreceptors suggests that gasocrine signaling is likely a widespread and fundamental biological phenomenon.

### Diversity of gasoreceptors that mediate gasocrine signaling

Gasoreceptors seem to be highly diverse in terms of the gases they sense, their cellular localization, their signaling domains, and the signal transduction pathways in which they can function (Figures 1E–I).

#### Diversity in gases sensed and cellular localization

Currently, proteins that act as gasoreceptors have been reported for O_2_, NO, CO, and ethylene in diverse organisms. Even mammalian N-methyl-D-aspartate (NMDA) receptors can be considered to exhibit duality as xenon gasoreceptor (Andrijchenko et al., 2015; Colloc’h et al., 2007; Zhou and Tajima, 2023). The majority of the gasoreceptors are in the cytoplasm, but some are also localized or functional in the cell membrane, endoplasmic reticulum, mitochondria and nucleus (Anbalagan, 2024c; Arnold et al., 1977; Binder, 2020; Delgado-Nixon et al., 2000; Horst et al., 2019; Kapetanaki et al., 2018; Murgo et al., 2023).

The question of whether volatile organic compounds (alcohols, esters, and etc..,) and receptors that sense them can be considered as ligands and gasoreceptors, respectively, in gasocrine signaling is one that needs to be debated. These compounds are released by various organisms in a manner similar to that of gaseous signaling molecules, and they can also be detected by specialized protein-based receptors and trigger physiological or behavioral responses (Pichersky and Gershenzon, 2002; McBride, 2016; Vlot and Rosenkranz, 2022; Pollock and Odom John, 2023).

#### Diversity of metal cofactors in gasoreceptors

To the best of my knowledge, most gasoreceptors require metal ions or cofactors for their gas binding. Although ethylene was one of the first gases identified as a signaling molecule, one of the first reported gasoreceptor was the NO-sensing soluble guanylate cyclase, which binds NO via the heme NO/oxygen (H-NOX) domain (Arnold et al., 1977; Horst et al., 2019). Heme NO/oxygen domain-containing proteins are found throughout the entire tree of life, even in some viruses (Wong et al., 2021). Similar hemoproteins have also been identified as CO and O_2_ gasoreceptors (Delgado-Nixon et al., 2000; Aono, 2017). In contrast, ethylene gasoreceptors contain ethylene-binding domains that bind ethylene via copper ions (Binder, 2020). However, it is likely that copper ion-binding proteins can sense other gases as well. For example, the O_2_-transporting protein hemocyanin found in various mollusks, marine and terrestrial arthropods can bind O_2_ (Felsenfeld, 1954; Hirota et al., 2008). This suggests the potential of additional gases sensed by Cu ion-based gasoreceptors. The presence of heme- and copper-ion-based metalloproteins that act as gasoreceptors warrants a reevaluation of the role of other metalloproteins as gasoreceptors (Kretsinger et al., 2013; Muok et al., 2019).

Many organisms express transporters for various metal ions, including zinc, molybdenum, nickel, and selenium, among others (Arguello et al., 2012; Nozoye et al., 2021). Zinc-based metalloproteins can even be considered potential H_2_S gasoreceptors (Flores et al., 2005). In addition to the existence of cellular metal ion import machinery, diverse organisms and cells can synthesize various metallic clusters, such as iron-sulfur, [Fe-Fe], [Ni-Fe] and [FeMo-co] clusters, among others (Peters et al., 2015; Burén et al., 2020; Braymer et al., 2021). However, it is unclear whether these clusters sense only the reactive species of gases or if they can also bind to gases directly (Aono, 2017). Such clusters are also present in enzymes such as nitrogenase that reduce atmospheric N_2_ to NH_3_. But whether some of the subunits in such N_2_-binding metalloproteins complex can be considered as N_2_-gasoreceptors remains unanswered (Sickerman et al., 2017). Overall, it is unclear how many other different metalloproteins can interact with gases *in vivo* and act as gasoreceptors.

#### Metal-cofactor independent gasoreceptors

The question of whether gasoreceptors can include metal-cofactor independent interactions is still up for debate. For example, the mammalian NMDA receptor can be considered as xenon gasoreceptor, as its interaction with xenon occurs directly at the aromatic amino-acid residues in a metal-cofactor-independent manner (Andrijchenko et al., 2015; Colloc’h et al., 2007; Zhou and Tajima, 2023).

But when considering such gas-interacting proteins, it is crucial to distinguish between interactions with freely diffusing gases and enzyme-mediated transfer reactions. For instance, NO-transferring metalloproteins are now referred to as nitrosylases and denitrosylases (Stomberski et al., 2019). Thus, NO-based S-nitrosation is more similar to an enzymatic transfer reaction than to the direct interaction of freely diffusing NO. Analogous to nitrosylases for NO, CO_2_ “fixing” carboxylases have been reported in photosynthesis-capable organisms (Stoffel et al., 2019; Bathellier et al., 2020). Meanwhile, proteins such as mammalian hemoglobin and ubiquitin can reversibly interact with freely diffusing CO_2_ through carbamylation at neutral N-terminal α-amino or lysine ε-amino groups (DiDonato et al., 1983; Linthwaite et al., 2021; Storz, 2018). These direct gas interactions support considering such proteins as gasoreceptors. For example, vertebrate hemoglobin, a nitrite reductase, could also be regarded as CO_2_ gasoreceptor in one-component signal transduction system if CO_2_-bound and O_2_-unbound hemoglobin can synthesize NO and trigger a cellular response (Perissinotti et al., 2008; Helms and Kim-Shapiro, 2013; Anbalagan, 2025a). Overall, gasoreceptors may include proteins that interact directly with gaseous molecules.

#### Diversity in signaling domains

In terms of signaling domains, gasoreceptors appear to be enzymes, transcription factors and ion channels (Kapetanaki et al., 2018; Murgo et al., 2023). To date, experimentally validated enzyme-based gasoreceptors include soluble guanylate cyclase, soluble adenylate cyclase, histidine kinases, and phosphodiesterases in diverse organisms (Arnold et al., 1977; Aono, 2017; Anbalagan, 2024c; Horst et al., 2019).

#### Diversity in signal transduction systems

Based on the classification of signal transduction systems (STSs) in prokaryotes, gasoreceptors seem to function in both one- and multi-component STSs (Buhrke et al., 2004; Ulrich et al., 2005). In co-component STS, a gasoreceptor can be a dimeric (or oligomeric) protein, where either the input domain or the output domains are shared between monomeric subunits. Examples include plant ethylene gasoreceptor kinases and mammalian nitric oxide gasoreceptor sGC (Binder, 2020; Horst et al., 2019). In addition to these STSs, gasoreceptors can also potentially function in proto- and split-component STSs which are evolutionarily ancient forms of signal transduction that has been proposed to predate one-component STS (Anbalagan, 2025a).

#### Can any protein be considered as gasoreceptor?

In theory, any protein whose activity is affected by direct gas interaction, regardless of its function, is a putative gasoreceptor (Anbalagan, 2025b). This raises the question of how potential gas-interacting proteins such as proteases or RNA-modifying enzymes can be considered as gasoreceptors when they are not typically considered to be enzyme-based receptor classes. Similar questions were asked when one of the first transcription factor-based estrogen receptors were reported. These receptors were non-membranal and non-enzyme-based, which challenged biochemists and receptor experts at the time (Moore, 2012). Another reason to rule out proteases as a receptors is the need for a molecular cascade to trigger transcriptional changes in the cell. However, as long as a molecular signaling events occurs inside or between cellular regions, organelles, or organelle-like condensates and triggers a response, it is a “cascade”, regardless of whether we consider it as such. The response does not have to be a message to the nucleus and its counter response. Responses could be even to other organelles or organelle-like elements within a cell.

Generally, a strict and restrictive criteria can be used to define receptors as proteins whose ligand-binding status-dependent signaling responses trigger a transcriptional changes in cells. However, even nuclei-less cells such as mature erythrocytes and platelets express receptors (Balabin et al., 2018; Tanneur et al., 2006). This challenges the need for nuclear response in receptor-mediated signaling mechanisms. Even the well known O_2_-transporting hemoglobin is proposed as an O_2_ gasoreceptor in split-component STS (Anbalagan, 2025a).

Finally, even small enzymes that are competitively inhibited by gases can be considered proto-gasoreceptor for those gases. Proto-gasoreceptors are evolutionarily-ancient form of gasoreceptors which are competitively inhibited by gas binding and lack additional allosteric sites. Such proteins may function in a proto-component STS (Anbalagan, 2025a). This rationale can similarly be extended to ion channels or transcription factors that lack allosteric sites, where their ion channel or transcriptional activity is competitively inhibited by gas-binding. Therefore, gasoreceptors can include proteins with any activity, not just kinases or other major classes of enzyme-based receptors (Anbalagan, 2024a; 2024c). However, such gasoreceptor must act as at least in one of the signaling systems: one-, two-, co-, multi-, split-, or proto-component STSs.

### Potential diversity in gas-sensing biomolecules

Although gasoreceptors are the best-known gas-sensing biomolecules, it remains unclear whether they represent the entire diversity of gas-sensing biomolecules across all cells or organisms. Gasocrine signaling may also occur via yet to be identified gas-sensing nucleic acids or other biomolecules. These include riboceptors, which can be composed of gas-sensing RNA-based riboswitches and ribozymes either individually or in combination (Anbalagan, 2024c). In principle, even the catalytic RNAs present within ribosomes could serve as prospective candidates for gas-sensing riboceptors (Mestre-Fos et al., 2020). Therefore, although the current definition of gasocrine signaling is restricted to gasoreceptor-based signaling events, it can be extended to non-protein-based gasoreceptors as well.

## Gasocrine hypothesis from the perspective of aerobic eukaryotic organisms

Gasocrine signaling encompasses signaling within organelles and cells, as well as signaling between different organisms and between abiotic gas-releasing materials, objects, and machines. Furthermore, just as the force of gravity acts throughout the entire planet, gasocrine signaling extends beyond individual cells and organisms to encompass entire ecosystems and the planet as a whole (Figures 1A–D). Given the potential diversity of gasoreceptors that mediate gasocrine signaling and the omnipresence of gases, a question arises: can an unifying theory in cell biology be developed?

For the sake of this discussion, I will first address the possibility of such a hypothesis for aerobic eukaryotic organisms that require O_2_ for its metabolism and survival. For O_2_-based inter-organismal gasocrine signaling to occur, O_2_ must first be synthesized and diffuse out of O_2_-producing organisms. Similar to experiments involving knocking out ligand-coding genes, knocking out all genes and genetic elements encoding O_2_-synthesis in all O_2_-generating organisms on Earth would likely lead to the cessation of O_2_-dependent aerobic life, though not necessarily all biotic life (Tan et al., 2024; West, 2022). This suggests that, for aerobic eukaryotic organisms, O_2_ is an inter-organismal signaling molecule and the message it conveys at physiological concentrations is simply “live,” irrespective of the biochemical means by which life is achieved.

Nevertheless, a strong counter argument is the fact that the role of O_2_ in aerobic eukaryotes is primarily in metabolism or as terminal electron acceptor in the mitochondrial electron transport chain (Cogliati et al., 2021; Storz and McClelland, 2017). In addition, O_2_ is used as a substrate by O_2_-dependent enzymes, of which more than 200 are reported in humans alone (Li et al., 2023). This raises the question: Which is the more important role of O_2_, is it as a signaling molecule and ligand for O_2_ gasoreceptors or as a metabolite and substrate for O_2_-dependent enzymes?

First, the number of metabolism-related enzymes that act as gasoreceptors is unclear. For instance, in the absence of O_2_, some of the mitochondrial electron transport chain subunits are competitively inhibited by gases. NO, H_2_S, and HCN can competitively inhibit cytochrome c oxidase (Complex IV), thereby blocking its ability to reduce O_2_ to water (Brown, 2001; Hanna et al., 2023; Poderoso et al., 2019; Zuhra et al., 2025). Thus, even the major metabolic enzyme cytochrome c oxidase can be considered as a derivative of a proto-gasoreceptor. But for a cell or a mitochondria is the lack of water a signal? It can be a signal, if it directly triggers a receptor-based response (or due to its role as a substrate). I previously proposed that water can potentially be sensed via gasoreceptors that exhibit duality as aquareceptors (Anbalagan, 2024d; Kim and Wang, 2016; Typolt and Filingeri, 2020). Numerous proteins in the mitochondria and a cell can be considered potential aquareceptors. Overall, these potential mechanisms suggest that gasocrine signaling events that initiate from O_2_-producing organisms extend to organelles, such as mitochondria, in aerobic eukaryotic organisms.

Second, experiments testing the role of O_2_ in metabolism will potentially affect the role of O_2_ gasoreceptor, and *vice versa*. Hemoglobin subunits have been reported to be localized in mitochondria, raising the possibility that they can act as a gasoreceptor in mitochondria as well (Anbalagan, 2025a; Brunyanszki et al., 2015; Reed et al., 2025). Therefore, based on the current literature, it is difficult to determine whether O_2_ is a major metabolic substrate or a major signaling molecule. Nevertheless, answering this question will require comparing a cell lacking all O_2_-dependent enzymes with a cell lacking all O_2_-gasoreceptors, whose identities are still unknown. The existence of eukaryotic organisms that do not require O_2_ for metabolism calls into question the importance of O_2_’s signaling role (Karnkowska et al., 2019; Al Jewari and Baldauf, 2023).

The identities of all the O_2_ gasoreceptors in various cell types are unknown. The presence of any O_2_-binding protein in a cell suggests the possibility of O_2_-based gasocrine signaling events likely occurring in that cell, based on potential gasoreceptors in one-, two-, multi-, split-, or proto-component STS. Since O_2_ role is essential for aerobic eukaryotic organisms, I propose that all the eukaryotic aerobic organisms require inter-organism gasocrine signaling.

## Gasocrine hypothesis–all cells require gasocrine signaling

As long as gasoreceptors and other gas-sensing biomolecules (riboceptors) can be identified, the “ligands” in gasocrine signaling include all biotically and abiotically produced gases including the inert gases (Dresp et al., 2019). It is unclear how many gases can cells or organisms can produce under naïve, physiological, stressed, pathological, or diseased conditions. Similarly, it is unclear how many environment-derived gases can diffuse into a cell under naïve or membrane-related injury conditions. Recently, reactive oxygen species-based methane (CH_4_) production has been identified in several model organisms, and even dihydrogen (H_2_) has also been identified as a signaling molecule in plants (Cheng et al., 2023; Ernst et al., 2022). However, the identity of methane- and dihydrogen- gasoreceptors in vertebrates or plants remains unknown (Aono, 2017; Ernst et al., 2022).

Due to the unlikelihood of biotic life in an absolute vacuum, the “seemingly” omnipresence of gases inside cells and in their microenvironments, diverse gas-synthesis pathways, as well as the role of gases as substrates for various enzymes in different organisms in the tree of life, I propose the gasocrine hypothesis: all cells require gasocrine signaling.

## Falsifation of gasocrine hypothesis

Historically, the field of biology has advanced due to serendipitous observations from experiments and the persistence of researchers in the face of challenges (Koshland, 2007; Matlin, 2022; Matlin et al., 2024). However, it also advanced when incoherent observations from nature or experimentation were connected under coherent, unifying postulates, dogmas, or speculative hypotheses that were later recognized as theories (Gunawardena, 2013). Even when Schleiden and Schwann first proposed the cell theory, their authority did not stop the spread of “free cell theory” or the spontaneous generation theory for several years (Danforth, 1870; Schwann, 1993). Even though Remak provided strong experimental evidence against the free cell theory, it required Virchow’s dictum “*Omnis cellula e cellula*” and forceful promulgation to definitively end the spontaneous generation theory (Lagunoff, 2002). This raises the question of how long it will take for the gasocrine hypothesis to become mainstream or to suffer the same fate as the theory of spontaneous generation of cells. To be validated, a hypothesis must be examined not only by experiments that support it, but also by those that can definitively contradict or falsify it. The lack of such robust falsification experiments led to the continued acceptance of spontaneous generation theory as well (Berche, 2012; “Spontaneous Generation,” 1867; Zwier, 2018). Therefore, amid decreasing science budgets, experiments investigating the gasocrine hypothesis should focus on falsifying it rather than proving them.

### All cells require gasocrine signaling

In my opinion, one of the various possible ways to disrupt gasocrine signaling is to ensure that a cell and its immediate microenvironment are devoid of any gases. Therefore, it must be prevented from receiving or producing gases. Additionally, it must be prevented from sensing any gases in any of the STSs (one-, two-, multi-, split-, proto- or others) (Anbalagan, 2025a; Capra and Laub, 2012; Ulrich et al., 2005). In this way, the cell will resemble a gas −/− and gas-sensing −/− double mutant. However, it is unclear how many genetic elements must be removed to even make a prokaryotic bacterium, such as *E. coli*, gas- and gas-sensing deficient.

There are three potential ways to falsify this postulate. The first experiment focuses on the gaseous molecules as signaling molecules. This experiment ignores the role of gaseous molecules in metabolism or as substrates for enzymes. The experiment involves finding a living cell that is devoid of any gaseous molecules and can live in an “absolute vacuum” or “absolutely degassed medium” environment. All other experimental parameters can be varied apart from the gases. A living cell could be a metabolically active cell or a dormant cell with minimal or no metabolic activity. However, if it is a dormant cell, it must exit dormancy under the experimental conditions. A potentially strong artifact of the experiment is distinguishing the effects of an absolute vacuum environment, such as low pressure and mechanical cellular damage, from the signaling role of gases. Whereas in the degassed medium, the medium must be degassed using a vacuum and not inert gases, such as argon or nitrogen. Moreover, degassing the medium does not necessarily mean that a cell will lose all its gaseous molecules; it may have a reservoir of gases inside it or associated with it. Thus, the “gas-free status” of a degassed medium cannot be ambiguous. Of the various parameters that can be altered during falsification experiments, temperature-induced liquid-to-gas phase changes and acoustic or vaporous cavitation may produce gaseous molecules. Therefore, any such conditions that will generate gases must also be excluded in experiments.

The second falsification experiment focuses on the gas-sensing elements in a cell. The goal is to find a living cell that lacks all gas-sensing factors. I am deliberately not using the terms “gasoreceptors” and “gas-sensing riboceptors” because I am unsure if these two will be the only gas-sensing biomolecules or factors that can function in one-, two-, multi-, split-, proto- or other STSs in a cell. A confounding factor in this experiment is the fact that receptors can exhibit duality and multimodality in sensing (Anbalagan, 2025b; Assadi-Porter et al., 2018). Knocking out a gasoreceptor will result in the loss of its gas-sensing role and its role in sensing other molecules or factors if the gasoreceptor exhibits duality. Another possibility is that it may have additional microenvironment-dependent adaptor or scaffolding roles. The same applies to gas-sensing riboceptors and other potential gas-sensing biomolecules. Thus, this approach risks pleiotropic effects. Nevertheless, studies using the knockout of coding or non-coding “genes” in microorganisms, plants and animal models are standard approaches that are widely accepted by the scientific community, despite such pleiotropic effects. However, it remains an open question how the systemic secondary effects can be ascribed only to gasocrine signaling.

The third falsification experiment involves finding a living cell that lacks both gases and gas-sensing elements. A true gas- and gas-sensing-free cell. However, describing such a cell without explaining its potential function will lead to various interpretations of the hypothesis. Therefore, the hypothesis that “all cells require gasocrine signaling” can be subdivided into three hypotheses: All living organisms composed of one or more cells require gasocrine signaling to sense, communicate, grow, and propagate. Gasocrine signaling mediated via gasoreceptor proteins (or yet to be identified gas-sensing riboceptors) is the most essential cellular and inter-organismal signaling. All cells and acellular entities arising from or replicating in pre-existing cells require gasocrine signaling.

### All living organisms composed of one or more cells require gasocrine signaling to sense, communicate, grow, and propagate

Although the falsification experiments will be largely similar to those listed in the previous section, the focus of the experiments will be on specific phenotypes rather than the overall state of a cell. The four different phenotypes include the cell’s ability to sense, communicate, grow and reproduce. Sensing can be for any biomolecule or factor, including, but not limited to, protons, photons, water, ions, lipids, amino acids, nucleic acids, proteins, temperature, gravity and radioactivity. Communication can occur via physical-, chemical-, electrical-, electromagnetic-, thermal-, light-, and acoustic means. However, such communication must not be an SOS-like last signal. Growth can occur via biosynthesis or volume expansion. Propagation can occur via sexual or asexual reproduction. Therefore, if a cell lacking gaseous molecules and/or gas-sensing biomolecules (e.g., gasoreceptors, gas-sensing riboceptors) can exhibit any of these functions in absolute vacuum or in an absolutely degassed medium, then this hypothesis will be falsified.

### Gasocrine signaling mediated via gasoreceptor proteins (or yet to be identified gas-sensing riboceptors) is the most essential cellular and inter-organismal signaling

This postulate prioritizes gasoreceptor-based gasocrine signaling over other signaling mechanisms in or between cells or organisms. However, gasocrine signaling triggered by other potential gas-sensing biomolecules, such as gas-sensing riboceptors, is not excluded (Anbalagan, 2024c). Therefore, the experiments will be focus on testing the importance or hierarchical nature of the signaling in comparison to other signaling mechanisms or pathways. Comparisons could be made with proton-, photon-, ion-, water-, amino acid-, peptide-, protein-, nucleic acid-, steroid-, lipid-, chemical temperature-, acoustic-, electrical-, physical-, mechanical-, visual-, and gravity-based signaling pathways, among others. Therefore, if a cell lacking gaseous molecules and/or gas-sensing biomolecules (e.g., gasoreceptors, gas-sensing riboceptors) can survive in an absolute vacuum or an absolutely degassed medium due to the functioning of its other ligands (or factors), receptors, and signaling pathways, then this hypothesis will be falsified.

### Acellular entities arising from or replicating in pre-existing cells require gasocrine signaling

This postulate highlights the importance of gasocrine signaling for the replication and biogenesis of acellular entities. The phenotype to be measured here is the potential ability of acellular entities to arise or replicate from a cell in the experiment. Acellular entities can include viruses, viroids, and biomolecular condensates amongst others (Flint et al., 2020). However, the cell itself must not become an acellular entity. Thus, if such acellular entities can arise from a cell or replicate in a cell lacking gaseous molecules and/or gas-sensing biomolecules (e.g., gasoreceptors, gas-sensing riboceptors) in an absolute vacuum or an absolutely degassed medium, then this hypothesis will be falsified.

### Falsification on synthetic- or proto-cell system

Falsification experiments must not only be performed on resilient organisms such as tardigrades or relatively less resilient immortalized or primary cell cultures. One easily observable experiment could involve a leaf from a plant, a fertilized chicken egg with or without its shell, or the giant unicellular, uninucleate organism *Acetabularia acetabulum* (Mandoli, 1998). However, as I mentioned earlier, a falsification experiment is not just about placing biological samples in an absolute vacuum or an absolutely degassed medium. It is also about engineering such samples that they cannot produce any gaseous molecule or alternatively do not express any gasoreceptors and gas-sensing riboceptors. To the best of my knowledge, there is a significant knowledge gap regarding the identity of all generated gases, gasoreceptors, gas-sensing riboceptors, and gas synthesis pathways, even in major model organisms (Aono, 2017). Without this knowledge, producing such an engineered cell or organism with absolute certainty of its properties will be challenging. Therefore, an alternative approach is to use a synthetic cell or proto-cell system.

Synthetic cell systems have been demonstrated to interact via physical and chemical modes (Mukwaya et al., 2021). Even protein- or nucleic acid-based membraneless biomolecular condensates can be considered as such systems. It seems that such synthetic cells could be relatively easily engineered, as bottom-up cells are constructed based on the scientists’ designs. Thus, if a synthetic cell lacking gaseous molecules and/or gas-sensing biomolecules (e.g., gasoreceptors, gas-sensing riboceptors) can sense, communicate, survive, grow, propagate, permit replication and release of acellular entity in an absolute vacuum or an absolutely degassed medium, then this hypothesis will be falsified for such a synthetic cell system.

### Ambiguity in falsification experiments

Despite their robustness, the above listed experiments have their own weaknesses. It is difficult to identify and attribute confounding factors or systemic secondary effects specifically to gasocrine signaling or some other signaling pathways or cellular processes. Nevertheless, the experiments are provided as a framework for researchers interested in them or in improving them. Another ambiguity is the role of gases as signaling molecules versus their role in cellular metabolism, which I discussed earlier. As an analogy, removing a fuel sensor in a not-so modern car will not stop the car from running. Similarly, removing the fuel is not the most appropriate experiment to determine whether the car will run. Moreover, a typical car cannot operate in an ocean of fuel or produce their own fuel. A more advanced car comes with multiple fuel redundancy systems. Removing one type of fuel or fuel sensor should not prevent it from running. Compared to a car, a cell is the product of a seemingly infinite number of generations, shaped by evolution and variable environmental factors. Modern cars have only undergone a relatively few iterations since first car was made, and some of it have hybrid options. So, despite the ambiguity of the falsification experiments, it is challenging to dismiss the gasocrine hypothesis without such experiments.

## Conclusion

The immediate implication of these postulates is that they supplement cell theory (Hyllner et al., 2015). However, the unintended implication is a perceivable challenge to the four pillars of biology: evolution, metabolism, genetics, and cell theory. My original intention was never to challenge these pillars or the underlying mechanisms. I started with a simple question: If there is a receptor for NO, is there a receptor for O_2_ (Anbalagan, 2024a)? Discussions and debates with some of the leading experts raised more questions. Rather than perceiving the postulates as a challenge to the four pillars of biology, perhaps understanding these pillars from a perspective of the pressure and challenges to it posed by gases and how gasoreceptors (and gas-sensing riboceptors) would have potentially mitigated those challenges is a more attractive alternative.

Moreover, the strong integration of environmental gaseous molecules into gasocrine signaling provides a different perspective on the origin of diseases, inter-organismal communication, consciousness and failure of majority of the drugs in clinical trials (He, 2023; Heindel and Vandenberg, 2015; Sun et al., 2022). For example, protein aggregates such as amyloid fibrils or Tau could be considered potential gasoreceptors unless all associated enzyme activities and gas binding are experimentally disproved. Similarly, consciousness in animals, plants, or ecosystems could be potentially be viewed as the sum of gasocrine signaling activities. In the case of drug failure in clinical trials especially for non-monogenic diseases or disorders, potential functional redundancies or a lack of homeostasis in gasocrine signaling may be an additional reason.

Even in the research field of host-microbe and host-pathogen interactions, a few major questions arise. For instance, it is becoming increasingly clear that the microbiome plays a role in diseases such as cancer and Alzheimer’s disease. This is because microbes or microbe-like elements have been observed not only associated with, but also inside, the cancer cells, as well as in neurons of deceased patients with Alzheimer’s disease post-mortem (Sepich-Poore et al., 2021; Che et al., 2024). In mice models of drug toxicity, the gut microbiome has been even linked to the effects of drug toxicity (Zimmermann et al., 2019). Similarly, certain bacterial species in zebrafish can colonize club cells in the skin and are responsible for the released alarm substance (Schreckstoff) and alarm response during physical injury (Chia et al., 2019). However, it is unclear whether the host cells-resident microorganisms listed in the above examples only exhibit gas-based quorum sensing or also exhibit gasocrine signaling with the host.

For the field of microbiology, NO-based signaling is recognized as one of the quorum sensing pathways that integrate bacterial density with environmental information (Heckler and Boon, 2019). During host-pathogen interactions, some pathogenic microorganisms release NO to regulate host processes and increase their own survival (Jedelská et al., 2021; Mix et al., 2021). However, while the intra- or inter-microbial NO-based signaling that regulates pathogen density can be considered quorum sensing, pathogen-to-host NO-based signaling and control of host cell density cannot. Thus, the term “gasocrine signaling” fills this gap.

The reason that O_2_–based microbial density regulation across distant ecosystems is not considered quorum sensing, despite the involvement of O_2_ gasoreceptors in microbial growth, is unclear. This leads to the challenging question: What percentage of quorum sensing is gasocrine signaling, and vice versa? If gaseous molecules such as O_2_ are not considered to act as quorum sensing molecules, then perhaps a new field of study, microbial gasocrinology, will emerge. This field would have implications not only for gas-mediated microbial signaling, but also for host-pathogen interactions and gas homeostasis across ecosystems or an entire planet. Additionally, due to the role of abiotic components in the potential gasocrine signaling-related feedback loops across ecosystems and the entire planet, ignoring the relevance to the Gaia hypothesis is difficult. Thus, it will be challenging to falsify the Gaia hypothesis without considering the potential role of gasocrine signaling in it (Boyle, 2025; Stolz, 2017).

Scientific theories have played a major role in advancing both biology and physics (Gunawardena, 2013). Unlike physics, where equations often provide deterministic explanations, major biological theories typically began with provocative and speculative hypotheses. The gasocrine signaling hypothesis remains highly speculative in its current state. Nevertheless, it offers a robust theoretical framework for researchers to explore open questions experimentally and to establish foundational studies in the emerging field of gasocrinology, especially in the context of a changing environment.

## Abbreviations

CH_4_, Methane; CO, Carbon monoxide; CO_2_, Carbon dioxide; H_2_S, Hydrogen sulfide; HCN, Hydrogen cyanide; O_2_, Oxygen or dioxygen; NO, Nitric oxide; sGC, Soluble guanylate cyclase; STS, Signal transduction system.
